# Supplementary material for: OpenSAFELY: Representativeness of electronic health record platform OpenSAFELY-TPP data compared to the population of England
Source: Wellcome Open Res. 2022 Jul 18;7:191. [Version 1] doi: 10.12688/wellcomeopenres.18010.1 (PMC9346309; doi:10.12688/wellcomeopenres.18010.1)
Supplement: Peer review history

# Peer review: Reviewer response for version 1

**DOI:** 10.21956/wellcomeopenres.19969.r51579

**Published:** 2022-08-02

**Authors:** Evangelos Kontopantelis

**Affiliations:** 1 Faculty of Biology, Medicine & Health, Division of Informatics, Imaging and Data Sciences, University of Manchester, Manchester, UK

This is a clearly written paper that described the characteristics of the TPP population, compared to national (England) estimates, mid-year 2020. I only have a few minor points to raise.

1. Explain that you created 5-year bands in the TPP patients, so they could be compared to ONS age groups.
2. Clarify IMD year when first described, and also explain for an international reader (e.g. what domains it includes, how many indicators e.g. see https://jech.bmj.com/content/72/2/140 for some useful references. ^2^
3. There is an issue with ghost patients, which hopefully is less of an issue following 2013 (there was an initiative to clear GP lists of ghost patients). Practices get paid on patient numbers so there is not much of an incentive to purge, which has led to some practices experiencing delays in death registrations. Although the authors do not seem to have many 120 year olds, it would be good to report if that has been checked. See more on the issue here: https://jech.bmj.com/content/72/6/532. ^2^ This also relates to the TPP coverage, which is almost certainly an overestimation.
4. The graphs look a bit basic but they convey the message.

Is the work clearly and accurately presented and does it cite the current literature?

Yes

If applicable, is the statistical analysis and its interpretation appropriate?

Yes

Are all the source data underlying the results available to ensure full reproducibility?

Yes

Is the study design appropriate and is the work technically sound?

Yes

Are the conclusions drawn adequately supported by the results?

Yes

Are sufficient details of methods and analysis provided to allow replication by others?

Yes

Reviewer Expertise:

Professor in Data Science and Health Services Research

I confirm that I have read this submission and believe that I have an appropriate level of expertise to confirm that it is of an acceptable scientific standard.

## References

[1] : Geographical epidemiology of health and overall deprivation in England, its changes and persistence from 2004 to 2015: a longitudinal spatial population study. *J Epidemiol Community Health* .72(2) : 10.1136/jech-2017-209999 140-147 10.1136/jech-2017-209999 29263178

[2] : Regional variation and predictors of over-registration in English primary care in 2014: a spatial analysis. *J Epidemiol Community Health* .72(6) : 10.1136/jech-2017-210176 532-538 10.1136/jech-2017-210176 29449351

# Peer review: Reviewer response for version 1

**DOI:** 10.21956/wellcomeopenres.19969.r51575

**Published:** 2022-07-21

**Authors:** Tom Fahey

**Affiliations:** 1 HRB Centre for Primary Care Research, Department of General Practice, Royal College of Surgeons in Ireland, Dublin, Ireland

Thank you for asking me to review this paper. It examines the representativeness of the OpenSAFELY-TPP dataset, referenced to the population of England using ONS data.

 The paper is clear and well written. The methods are appropriate and the conclusions are consistent with the data presented. OpenSAFELY-TPP is broadly representative in terms of deprivation (IMD), age, gender, ethnicity and cause of death. These are important findings in relation to generalisability/external validity of the data and any subsequent papers that will use OpenSAFELY-TPP.

Is the work clearly and accurately presented and does it cite the current literature?

Yes

If applicable, is the statistical analysis and its interpretation appropriate?

Yes

Are all the source data underlying the results available to ensure full reproducibility?

Yes

Is the study design appropriate and is the work technically sound?

Yes

Are the conclusions drawn adequately supported by the results?

Yes

Are sufficient details of methods and analysis provided to allow replication by others?

Yes

Reviewer Expertise:

Primary care epidemiology- observational, RCTs and systematic reviews

I confirm that I have read this submission and believe that I have an appropriate level of expertise to confirm that it is of an acceptable scientific standard.
